# Mechanisms of Inhibition of Quorum Sensing as an Alternative for the Control of *E. coli* and *Salmonella*

**DOI:** 10.3390/microorganisms10050884

**Published:** 2022-04-23

**Authors:** Esmeralda Escobar-Muciño, Margarita M. P. Arenas-Hernández, M. Lorena Luna-Guevara

**Affiliations:** 1 Posgrado en Microbiología, Centro de Investigación en Ciencias Microbiológicas, Instituto de Ciencias, Benemérita Universidad Autónoma de Puebla, Ciudad Universitaria, Puebla C.P. 72570, Pue, Mexico; esmeeem2014@gmail.com; 2 Colegío de Ingeniería en Alimentos, Facultad de Ingeniería Química, Benemérita Universidad Autónoma de Puebla, Ciudad Universitaria, Puebla C.P. 72570, Pue, Mexico

**Keywords:** Gram-negative bacteria, quorum sensing, QSIs, QSI resistance

## Abstract

Quorum sensing (QS) is a process of cell–cell communication for bacteria such as *E. coli* and *Salmonella* that cause foodborne diseases, with the production, release, and detection of autoinducer (AI) molecules that participate in the regulation of virulence genes. All of these proteins are useful in coordinating collective behavior, the expression of virulence factors, and the pathogenicity of Gram-negative bacteria. In this work, we review the natural or synthetic inhibitor molecules of QS that inactivate the autoinducer and block QS regulatory proteins in *E. coli* and *Salmonella*. Furthermore, we describe mechanisms of QS inhibitors (QSIs) that act as competitive inhibitors, being a useful tool for preventing virulence gene expression through the downregulation of AI-2 production pathways and the disruption of signal uptake. In addition, we showed that QSIs have negative regulatory activity of genes related to bacterial biofilm formation on clinical artifacts, which confirms the therapeutic potential of QSIs in the control of infectious pathogens. Finally, we discuss resistance to QSIs, the design of next-generation QSIs, and how these molecules can be leveraged to provide a new antivirulence therapy to combat diseases caused by *E. coli* or *Salmonella*.

## 1. Introduction

Quorum sensing (QS) is a process of cell–cell communication for bacteria such as *E. coli* and *Salmonella* that cause foodborne diseases, which consists of the production, release, and detection of signaling molecules known as autoinducers (AIs) that take place in response to changes in the bacterial population density. An increased local AI concentration causes shifts in the bacterial gene expression. These coordinated events allow microorganisms to respond to the bacterial environment, promoting their adaptation and ensuring their survival [1]. In addition, a relatively new concept of QS, “socio-microbiology”, has been introduced to explain some cooperative associations between bacteria, while others are antagonistic, resulting in complex behaviors [2,3]. There are reports of new autoinducer molecules and QS receptors, along with various ways to interrupt and inhibit signaling pathways to control bacterial pathogens [2,3,4].

Among the bacteria associated with foodborne illnesses are *Listeria monocytogenes*, *E. coli*, *Shigella*, *Salmonella*, and *Staphylococcus*; however, *Salmonella* and *E. coli* have been associated with increased outbreaks due to the consumption of contaminated raw vegetables [5]. These bacteria are clinically relevant and multidrug-resistant and have caused several cases of extra-intestinal diseases [6]. Several of the pathogenicity mechanisms of *E. coli* and *Salmonella* enterica subsp. *enterica*, ser Enteritidis and Typhimurium, are related to QS [7,8].

Pathogenic *E. coli* bacteria possess five principal QS systems, categorized as (i) AI-2 signaling produced by the enzyme LuxS, (ii) SdiA (suppressor of cell division/inhibition) signaling, a transcriptional regulator of the LuxR homolog receptor for homoserine lactone, (iii) the AI-3/epinephrine/norepinephrine signaling pathway involved in host-bacteria communication, (iv) indole signaling, mediated by the self-produced effector indole, and (v) Extracellular Death Factor (EDF) signaling, conveyed by a self-produced peptide that triggers the activation of toxin-antitoxin systems [9]. In general, QS in *E. coli* participates in regulating of virulence genes related to biofilm production, mobility, the type III secretion system (T3SS), toxicity, and the production of curli [10]. QS in *Salmonella* species is involved in the regulation of the pathogenicity island SPI-1 (the invasion phenotype), the expression of the flagellar genes, the *pefI-srgC* plasmid operon that regulates the genes *rck* (resistance to complement killing), and *srgE * (sdiA-regulated gene E) that independently encode an outer membrane protein RcK T3SS-1, which is involved in the Zipper invasion mechanism and a T3SS-1-dependent effector protein of unknown function, respectively [11,12,13].

For more than 20 years, there have been reports of a great quantity of Quorum Sensing Inhibitors (QSIs) as the chemically synthesized QSI derivatives of halogenated furanone, a compound obtained from red algae *Delisea pulchra*, which was initially tested on pathogens such a *Vibrio* and *Pseudomonas*. Strong inhibition of the QS mechanism has been found to prevent the transcription of virulence genes and the synthesis of autoinducers through the union of furanone with the QS transcriptional regulator [14,15]. Proctor et al., in 2020, reported that some halogenated furanones act as antagonists of QS and have been studied due to their ability to inhibit the QS of Gram-negative bacteria. Moreover, crystallographic and molecular docking studies have been used for the discovery of several QSI molecules [16,17]. In all studies, the ability of QSIs to attenuate various pathogenic microorganisms, including *E. coli* and *Salmonella*, has been highlighted [18,19,20].

In addition, a diverse family of QSIs derived from QS molecules, such as Acyl-Homoserine Lactone (AHL), AI-2, and AI-3, has been developed and obtained by research phases in vitro and in vivo [10,21,22,23,24]. In this work, the studies carried out on the QSIs in a period from 2005 to 2021 were considered.

It is also believed that QSIs target bacterial cell–cell signaling and coordinated activities required for infections. The QSI therapies that block bacterial QS can blind the pathogens rather than kill them. Therefore, QSI therapy can be used for treatment and causes less selective pressure to create resistant microbes [25]. As can be seen, the study of the possible mechanisms of evolution of these pathogens is important to determine the bacterial resistance to QSIs. Moreover, the status of QSIs is still in its investigative phase; pharmaceutical companies are currently developing research for the applications of QSIs for subsequent FDA (Food and Drug Administration) approval [26]. Current studies mention the strategy of combining antibiotics with QSIs, thus observing synergistic effects with antimicrobials in model bacteria such as *P. aeruginosa* and *B. cenocepacia*. In other research, the susceptibility to certain antibiotics such as tobramycin supplied with QSI has been increased in resistant and tolerant microorganisms such as *P. aeuroginosa* [27]. QSIs’ use with garlic extracts has also been observed to improve their anti-inflammatory and antimicrobial properties, while the use of QSIs with carbonates avoids the formation of biofilm on teeth [28].

In this work, we review the types and action mechanisms of QSIs, strategies used for their study, discuss how these molecules can be leveraged to provide a new antivirulence therapy against bacterial pathogens such as *E. coli* and *Salmonella*, and finish by outlining limitations such as resistance to QSIs and prospect.

## 2. The QS System Based on Autoinducer-2 (AI-2) in *E. coli* and *Salmonella* spp.

**Figure 1:**
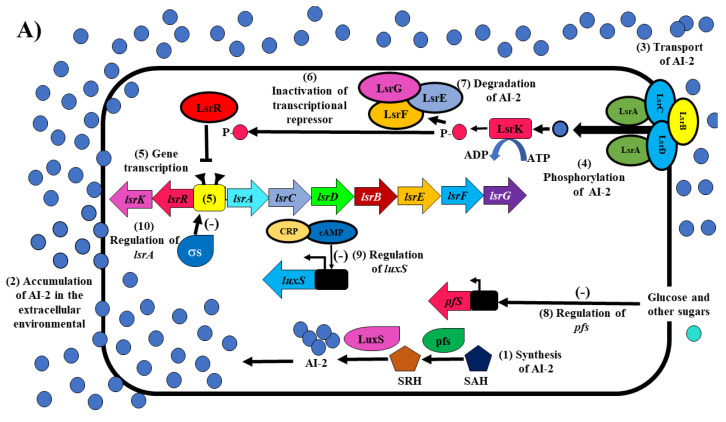

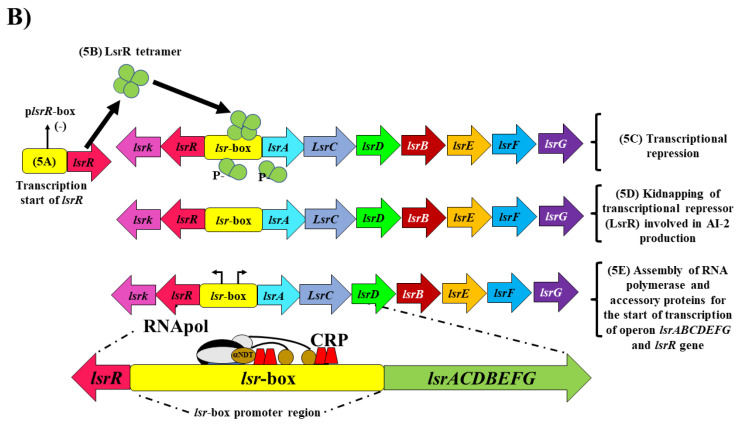
QS model in *E. coli* and *Salmonella* sp. (**A**) The mechanism of QS is based on AI-2. (**B**) The role of LsrR-AI-2-P as a transcriptional regulator of the *lsrACDBEFG* operon and the *lsrK* and *lsrR* genes. For more details see the text. The figures were based on information from [34,35,36].

Biosynthesis of AI-2 occurs through the methyl activation cycle (CAM), which several enzymes including AI-2 synthase (LuxS) participate in, which is encoded by the *luxS* gene. The LuxS substrate is S-ribosyl homocysteine (SRH), which is utilized by LuxS to produce homocysteine and DPD (4,5-dihydroxyl-2,3-pentanedione), which can be spontaneously cycled to form AI-2 as a final product (Figure 1A) [10]. AI-2 is synthesized by the 5′-methylthioadenosine/S-adenosylhomocysteine nucleosidase (Pfs) enzyme, according to its function in the methyl activation cycle (CAM) in the synthesis of AI-2; the main function of Pfs is to catalyze the irreversible cleavage of the glycosidic bond in both 5′-methylthioadenosine (MTA) and S-anhydro ribosyl-L-homocysteine (SAH) to adenine, and the corresponding thioribose, 5′-methylthioribose, and S-ribosylhomocysteine, respectively (Figure 1A, step 1) [29].

The import of AI-2 in *E. coli* and *S. typhimurium* occurs through a protein complex (LsrACDBEFG), which shows homology with some Gram-negative sugar transporters [30]. The signaling mechanism is based on the binding of AI-2 to the periplasmic protein receptor LsrB, initiating intracellular transport of AI-2 through two transmembrane proteins (LsrC and LsrD) and an ATPase protein (LsrA), which provide the energy necessary for the transport of AI-2 to the cytoplasm in the bacterial cell. In the cytoplasm, the LsrK protein kinase phosphorylates the AI-2 molecule, converting it to AI-2-P [10]. In the absence of AI-2-P, the expression of the *lsrACDBEFG* operon is repressed by the LsrR protein regulator. Furthermore, the function of AI-2-P is to capture the LsrR regulator protein from the promoter site to form a complex with LsrR-AI-2-P, causing the derepression of the *lsrACDBEFG* operon. Finally, the role of LsrR is to regulate and control the expression of the LsrR regulator protein and various genes in response to AI-2-P [10,31].

Subsequently, the AI-2 cycle is closed by the participation of three degrading proteins (LsrE, LsrF, and LsrG) that prevent the accumulation of AI-2-P, converting it to 3,4,4-trihydroxy-2-pentanone-5-P (P-TPO), and finally degrading it to phosphoglycerate and other small molecules produced in lower concentrations [32]. Figure 1B shows the role of LsrR as a transcriptional repressor protein (tetrameric form) and the role of AI-2-P when it binds to the transcriptional regulator protein LsrR (dimeric form). Subsequently, the RNA polymerase is posed and assembled at the promoter site indicated by the *lsr* box, propitiating the activation of the operon *lsrACDBEFG*, the production of biofilm, mobility, the activation of the type III secretion system, and the production of curli in *E. coli* and *Salmonella* species [10,33].

## 3. Inhibition of Quorum Sensing

There are different strategies to silence or inhibit bacterial QS, such as (i) inhibition of AI biosynthesis in Gram-negative bacteria by blocking AI-2 synthase, (ii) QS signal degradation in the extracellular environment by Quorum-Quenching (QQ) enzymes such as AHL-lactonase, oxidoreductase, and acylases, (iii) receptor blockage or interference with the AI/receptor complex, (iv) attenuation of the QS signal due to a complex formation between AI and molecular imprinting polymers (IMPs), or (v) degradation of enzymes that interfere with cell–cell communication, leading to the active control of the AI-2 concentration or availability [37,38,39,40,41].

## 4. Types of Inhibitors of QS

QSIs are natural or artificial ligands that bind to QS regulators; these molecules can be either pure or weak agonists, which effectively compete with the autoinducer molecules by binding with the transcriptional protein regulator of QS [42]. Recent investigations were related to the development of synthetic inhibitors involved in the production, perception of, and response to AI-2, and interaction with the LuxS, LsrB, LsrR, QscE, and SdiA regulator proteins. Specifically, studies with LsrR and SdiA transcriptional regulators from *E. coli* and *Salmonella* have been reported [43,44].

### 4.1. Natural Inhibitors

Important inhibitors have been found from the use of bacterial biosensors and molecular techniques. The main sources of these QSIs are some fruit extracts (blackberries, blueberries, vanilla extracts, and citrus), herbs (rosemary and turmeric), spice oils (garlic, cloves, and cinnamon), and phenolic compounds from plants. The latter compounds include benzoates, phenylpropanoids, stilbenes, flavonoids, gallotannins, proanthocyanidins, coumarins, and terpenes (monoterpenes, diterpenes, triterpenes, and sesquiterpenes) [45,46,47]. Some naturally occurring QSIs in *E. coli* and *Salmonella* species are described in Table 1.

### 4.2. Synthetic Inhibitors

Synthetic inhibitors are recognized as an alternative and attractive strategy for bacterial control practices applied in industrial settings. Instead of eliminating bacteria with conventional sanitizers, the blocking of cell–cell communication might inhibit the expression of virulence phenotypes, with a smaller likelihood of resistance development [55]. Several synthetic QSIs and their effects on bacterial infections are listed in Table 2; some inhibit *E. coli* and *Salmonella* by blocking QS regulators such as LsrR, SdiA, and QseC [10,21,40,56,57,58,59,60].

## 5. Mechanisms of QS Inhibition

Both for *E. coli* and *Salmonella* spp., the anti-virulence strategy proposed consists of the interference with QS through different means, such as the search for and design of QSIs that block the synthesis of AI-2, the receptor protein LsrB, the transcriptional repressor of QS (LsrR), or the LuxR solo regulator (SdiA) [10,22,43,45,60]. The most studied mechanisms of QS inhibition are described below.

### 5.1. Inhibition of AI-2 Synthesis

Blocking the main enzymes of the AI-2 synthesis pathway, methyltransferase and 5′-methylthioadenosine, has been achieved through the use of inhibitors of intermediary molecules of the cycle of the methyl activation pathway: for example, S-anhydro ribosyl-L-homocysteine (SAH) and S-homoribosyl-L-cysteine (SRC). In this way, some inhibitors of the Pfs enzyme have been reported [39,45].

**Figure 2:**
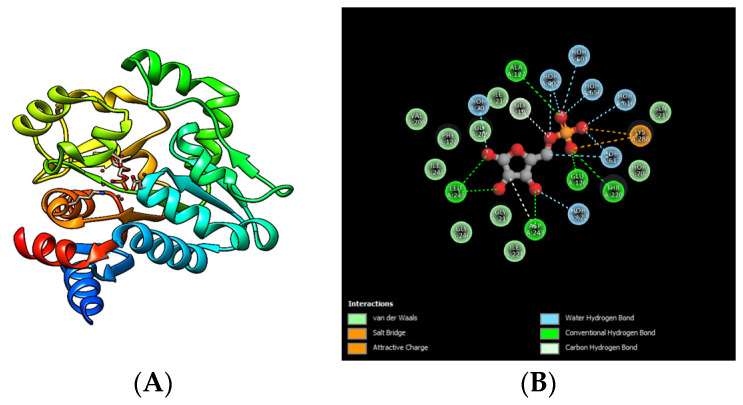

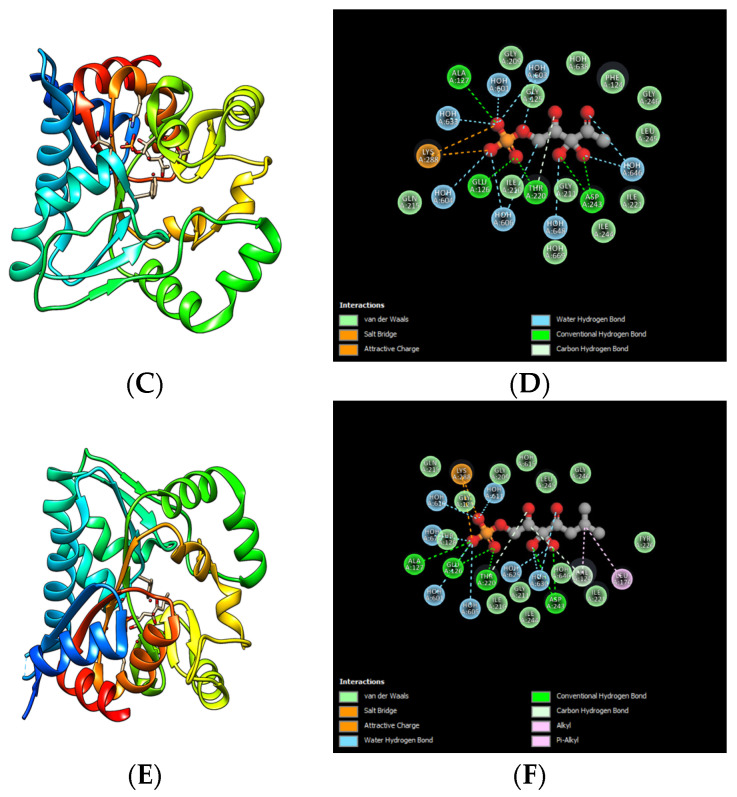
Molecular docking of QSIs in *E. coli*. (**A**) Transcriptional repressor LsrR in the interactions with A1-2-P in the ribbon model. (**B**) Interactions of the amino acids representative of the LsrR regulator with the functional groups of AI-2. (**C**,**E**) Interactions of LsrR with the inhibitors D5P and D8P, respectively. (**D**,**F**) Interactions of the amino acids representative of the LsrR regulator with the functional groups of the inhibitors. The images were reproduced from the study of Ha et al. (2014) using the PDB, then reproduced in PyMOL software (TM) version 2.3.4 [39].

The interaction between AI-2-P and the LsrR regulator protein is described by molecular docking in *E. coli*, where the hydrogen bridging interactions of the amino acids from the catalytic site of LsrR (PDB: 4L51), and the natural ligand, are also shown (Figure 2A). The principal interactions between AI-2-P and LsrR are found in the cyclopentane ring of AI-2, which interacts with the amino acids Gly 209, Asp 243, and Leu 245 of LsrR. The PO_4_^−2^ group of AI-2-P can interact with the amino acids Lys 288, Ala 127, Thr 220, and Glu 126 of LsrR (Figure 2B) [39]. On the other hand, using molecular-docking studies and bioinformatics tools, we can represent the interactions of the synthetic inhibitor 2S-2,3,3-trihydroxy-4-isopentyl dihydrogen phosphate (D5P) (PDB: 4L4Z) with the amino acids Glu 126, Thr 220, Lys 288, Ala 127, and Asp 243 of LsrR (Figure 2C,D). Meanwhile, the interaction of another synthetic inhibitor, 2S-2,3,3-trihydroxy-6-methyl-4-oxoheptyl dihydrogen phosphate (D8P), with LsrR (PDB: 4L50) involves amino acids Ala 127, Lys 288, Glu 126, Thr 220, Asp 243, and Phe 124 (Figure 2E,F) [39]. In this way, the QSIs simulate the competence with the signaling molecule for receptor binding [39]. This demonstrates that the use of molecular docking is a tool for in silico selection and experimental validation of FDA-approved drugs as anti-quorum sensing agents [26].

### 5.2. Inhibitors of the Incomplete QS System

**Figure 3:**
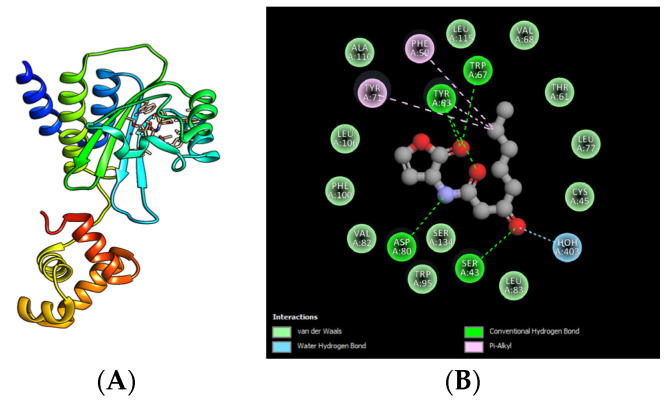

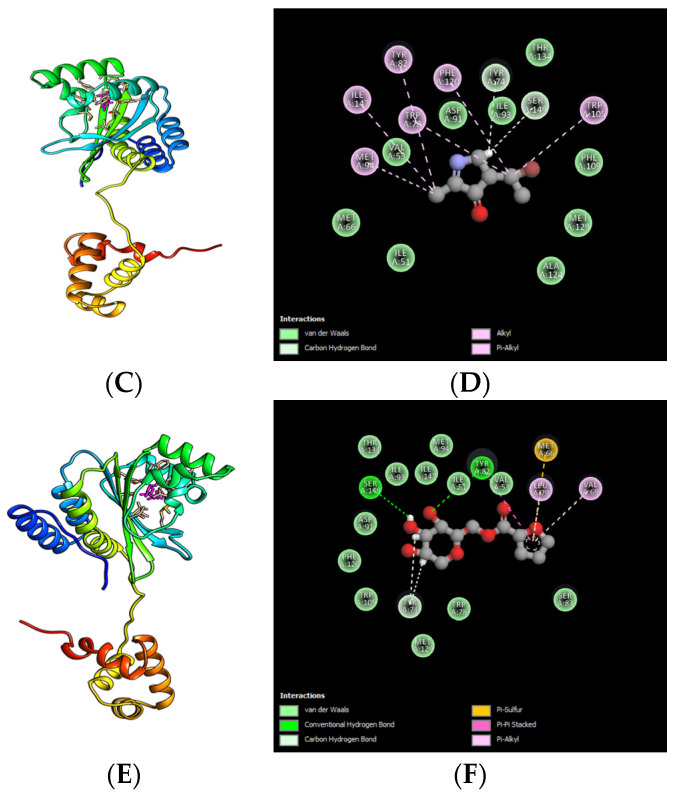
Molecular docking of QSIs in SdiA. (**A**) SdiA protein regulator and C_8_-AHL, represented by the ribbon model. (**B**) Molecular interactions of the amino acids belonging to the SdiA regulator and the regulator with the functional groups of the C_8_-AHL autoinducer. (**C**,**E**) Interactions of SdiA with the inhibitors BL39R1 and fructose-furoic acid ester, represented by the ribbon model. (**D**,**F**) Interactions of the amino acids representative of the SdiA regulator and the functional groups of the inhibitors BL39R1 and fructose-furoic acid ester. The representations were obtained from PDB data reviewed in 2020, then reproduced in PyMOL software (TM) version 2.3.4, or were adapted from different molecular docking studies [37,38,40].

The inhibitors of transcription of the SdiA regulator protein in *E. coli* and *Salmonella* species attenuate the expression of virulence factors by blocking the binding of AHLs to transcriptional regulator SdiA (Figure 3A,B). A detailed example of the interaction of the natural ligand C_8_-AHL with the amino acids Ser 43, Tyr 63, Trp 67, and Asp 80 from SdiA (PDB: AY17) is described in *E. coli*. Meanwhile, the inhibitor 7-(1-bromoetil)-3, 3-dimetil-bicyclo [4.1.0] heptan-2-one (BL39R1) interacts through hydrogen bridges with different SdiA amino acids (Phe 63, Tyr 75, Tyr 67, and Val 86) (Figure 3C,D). In addition, the interaction of the inhibitor fructose-furoic acid ester with the amino acids Ser 43, Tyr 63, Trp 67, Tyr 71, and Asp 80 from the protein regulator of SdiA showed a π-π interaction with Trp 95, as can be observed in Figure 3E,F [37,38,40].

## 6. Strategies Used for the Study of QSIs

Certain methodologies used to analyze the QSIs are as follows: (1) The study of homologous LuxI/R genes that participate in the QS of *E. coli* and *Salmonella*, (2) purification of natural extracts or the design of libraries of QSI synthetics, (3) use of QS biosensors to detect possible inhibitors of autoinducer molecules, (4) determination of the average inhibitory concentration (IC_50_) with anti-QS compounds (organic or synthetic), (5) observations on the inhibition of some phenotypes related to virulence in pathogenic bacteria, (6) confirmation of the in vitro effect of QSI through the cloning transcriptional regulator of QS in a bacteria model, and (7) use of molecular docking for the analysis of possible mechanisms of QS inhibition, through the interaction of the organic or synthetic compounds with the transcriptional regulator or repressor of QS. These tools allow us to understand the inhibition mechanisms of QS and subsequently propose anti-QS therapies [61,62,63,64,65].

## 7. Studies of QSI Uses

Currently, several inhibitors have been approved by the FDA which have been used as antivirulence agents targeting the QS detection system of bacterial pathogens [66]. Some inhibitors have also been approved with pharmacological activity for use in clinical trials in vitro, using biosensor screening in silico experiments, and in vivo by a mouse model [26,67,68].

QSIs represent a new generation of antimicrobial agents with applications in human and veterinary medicine, agriculture, aquaculture, and biotechnology. Several QSIs are produced by companies such as QSI Pharma A/S, LEO Pharma microbia, and 4SC AG, all of them interested in developing anti-QS molecules [45,69,70]. Studies are focused on inhibiting the communication between bacteria and reducing bacterial pathogenesis. The results are very promising and these molecules can be industrially synthesized [41].

There are few studies in *E. coli* and *Salmonella* sp. about the action of QSIs on the participation of the LsrB, LsrR, and SdiA proteins. However, the synthetic inhibitor known as thiophenone (TF101) is a good competitive AI-2 inhibitor that antagonizes the AI-2 receptor protein (LsrB); this relationship has been verified by in silico studies [10,37,44].

Other studies of QS inhibition through in vivo models carried out with *Caenorhabditis elegans* and *Galleria mellonella* have allowed the exploration of the inhibitory activity of baicalin hydrate, cinnamaldehyde, hamamelitannin, tobramycin, vancomycin, and clindamycin, which causes a decrease in the formation of biofilm, enhances the sensitivity of Gram-negative pathogens to antibiotics, and increases the host survival rate after infection [22]. Other studies have confirmed that synthetic inhibitors like the furanone derivatives have useful pharmacological activities as anti-inflammatory, anti-cancer, and anti-microbial agents that are effective in the reduction of the AI-2 synthesis pathway and for bacterial biofilm formation in animal models [64,71,72]. Rui et al., in 2012, reported that the inhibitor (4S, 5R)-DHD presented biological activity at low concentrations, showing a strong antagonistic effect with the LsrR receptor of *E. coli*, concluding that the use of this inhibitor is a new way to manipulate the QS. Moreover, QSI furanone types have been used in different medical materials like catheters, coatings, urinary catheters, and medical devices for the inhibition of bacterial QS [67,73]. An example is furanone, which is used in combination with other compounds for coated catheters, being effective against pathogenic bacteria and controlling the infection for two months [74]. In specific cases of uropathogenic *E. coli* (UPEC), the use of furanone decreases the establishment of catheter-associated urinary tract infections, which may be considered for future research on the design of new drugs based on the furanone structure for evaluation as anti-biofilm catheter-coating agents in combination with other natural inhibitors, to test the synergistic effect, and evaluate the activation and inactivation of genes regulated by QS [75]. For the *Salmonella* genus, the use of QSIs such as as furanone and derivatives has been considered by food microbiologists to inhibit the virulence related to QS; it has been demonstrated during in vitro experimentation that there is a decreasing effect on the biofilm, some virulence factors, and AI-2 production [76]. These results are of interest to the food industry to resolve the contamination of food contact surfaces, such as tables, kitchen utensils, walls, floors, and machinery, highlighting QSIs’ possible applications in the formulation of disinfectants that help to preserve human health [77,78].

In other cases, resistance to certain natural or synthetic QSIs has been demonstrated in Gram-negative strains through the adaptation of bacterial strains, by activating resistance mechanisms, and promoting conjugative transfer and mutation. This effect has been confirmed in some QSIs, facilitating plasmid RP4 conjugative transfer, achieved by binding with the SdiA protein to regulate pilus expression and by interacting with the LsrR protein to increase the SOS gene expression and induce gene mutation [75,79,80]. In addition, certain compounds have been proposed as QSIs that can influence the expression of virulence genes acting as mimicking compounds to the autoinducer molecules, causing an effect contrary to the inhibition of QS mechanisms. The consequences are to influence or trigger gastrointestinal diseases or urinary infections in susceptible patients [75].

At the same time, many in vitro studies on QSIs have been performed in several cell lines such as Caco-2, kidney carcinoma cell line A498, and human colonic cell line HT-29, and in animal models such as mice (tissues of lung, liver, spleen, and kidney cell line C57BL/6), with good results in blocking the QS mechanism [10,40,52,57]. Studies on cell lines help evaluate the effect of a QSI before carrying out a study on humans for its subsequent validation by the FDA during the clinical phases at scale to confirm its therapeutic relevance in the future [26].

Finally, the inhibition of QS using FDA-approved drugs available on the market is a promising strategy for inhibiting virulence factors without affecting the normal microbiota, which could be used as a therapeutic alternative to traditional antibiotics. These QSIs can be used as a bacterial control method and provide useful information for the design of new QS modulators [68,73].

## 8. Resistance to QSIs

Unlike antibiotics, QSIs can have an antimicrobial effect without generating microbial resistance; as an example, *Chromobacterium violaceum* was evaluated with natural extracts obtained from vanillin [81,82]. Current efforts in QQ research are dedicated to expanding the discovery of new extracts and molecules with anti-virulence properties that are useful against important multidrug-resistant Gram-negative bacteria. Additionally, the synergistic activity of some QSIs with conventional antibiotics has been reported [83,84]. Initially, antibiotics were reused, causing an effect on bacterial QS; in some cases the antibacterial activity decreased growth, and QS inhibition was demonstrated. An important difference between antibiotics and QSIs observed by other authors in this field of research denotes that, unlike classical antimicrobials, QSIs inhibit virulence rather than bacterial growth, thus minimizing the possibility of generating resistance [24,81,85]. In addition, there was rigorous research about the synergistic effect of antibiotics and QSIs on biofilm-producer bacteria under in vitro and in vivo conditions in a mouse model; reduction of the bacterial load, increased bacterial susceptibility, and survival of the host were observed [86].

On the other hand, studies about the effect of thiolactone analogs have been evaluated with transcriptional regulator homologs of QS such as LuxR, LasR, and TraR, which showed antagonistic activity in the QS of some bacterial pathogens [87]. Similarly, other QSIs derived from AI-2 have been implemented in pathogens such as *E. coli* and *Salmonella* species, demonstrating a decrease in virulence [73,88,89]. All this provides valuable information for the design of the next generation of chemical tools to study and manipulate QS systems in bacterial pathogens [90]. 

*E. coli* and *Salmonella* have mechanisms that evade the action of QSIs because these bacterial species can recognize different autoinducer signals from QS by several regulation circuits, thus maintaining the regulation of transcription of virulence genes related to biofilm production or toxicity. These virulence genes are regulated by different QS systems, such as a complete system of QS (LuxS/LsrR), an incomplete system of QS (SdiA), and indole signaling [37,40]. System secretion type III (SST3) is regulated by the membrane-bound sensor kinase (QseC), which is activated by three optional QS molecules (AI-3, epinephrine, and norepinephrine), demonstrating the variability of detection systems by different QS molecules and the strategies of competence by other inhibitors of QseC to become a resistant strain to QSI [91]. Moreover, the QS system that responds to indole in *E. coli* and *Salmonella* regulates the plasmid stability, the expression of virulence factors, the resistance to antibiotics, and the adaptation of bacterial cells when there is a nutrient-poor environment. At the same time, there is competition between the indole and autoinducer molecules for the AHL domain-binding of the SdiA transcriptional regulator [51,92]. In this way, indole reduces bacterial virulence and competes with other inhibitors, causing bacterial resistance [51,92]. The bacteria under environmental stresses can evade the QSIs by retarding QS signal synthesis, depending on growth phases [93]. Moreover, the bacteria present selective pressure to the QSIs, decreasing with it the risk of developing resistance to these components, but in this regard, opinions vary according to observations with several bacterial models and QSIs [61].

The multiplicity of QS systems is likely to provide bacteria with opportunities to evolve and develop resistance to QSIs. Thus, bacteria can switch pathways to evade the action of QSIs [93]. In other cases, bacteria manifest resistance to antibiotics, toxins, heavy metals, and biocides by exploiting their efflux pumps. Some antibiotics can alter membrane permeability, which can also be instrumental in disturbing the efflux of QS signals [93].

There are few examples in the literature, but some provide valuable information such as the QSIs that block the transcriptional regulator LuxR and their homologs, such as the transcriptional regulator LsrR, in bacterial strains of clinical interest that are resistant to furanone C-30. No significant effect on growth has been observed; the main effects were that bacteria survived and developed resistance to the inhibitor [24]. At the same time, some amino acid residue mutations of the LuxR protein regulator in Gram-negative bacteria have been reported, increasing the affinity to the inhibitor and causing resistance [24,94,95]. There is additional evidence of bacterial resistance to treatments with QSIs through the inhibition of the transcriptional regulator LuxR that binds to AI-1. Furthermore, it has been found that the LuxO inhibitor acts through an uncompetitive mechanism by binding to the LuxO-ATP binding site and thus forming a complex to inhibit ATP hydrolysis. This was validated by mutational studies of the Walker B motif (ATP-binding box) of the LuxO protein. Each of the three mutants in LuxO was found to be resistant to synthetic inhibitors based on the structure of the autoinducer CAI-1 [96]. 

At the same time, the QS system based on the transcriptional regulator LsrR and two promiscuous receptor proteins (LsrK and LsrB) exhibits a response to treatment with QSIs. This has been verified with several alkyl inhibitors based on the DPD molecule when investigating the effect on Gram-negative bacteria of different chain lengths of the inhibitory molecule. It has been discovered that incorporation of the DPD analog inhibitor increases the repression of the *lsrACDBFG* operon (which encodes for AI-2 transport proteins in the cellular membrane) and the *lsrK* (which encodes for an AI-2 kinase in the cytoplasm) and *lsrR* (which encodes for the regulator of QS in the cytoplasm) genes by employing phosphorylation kinetics [43].

Bacteria become resistant to QSIs through regulation of their QS system to promote the expression of resilience to environmental and enhanced production of QS signals, which enable bacteria to produce virulence factors [15]. One treatment used is to modify the microenvironmental conditions during QSI protocol therapy to combat the resistance phenomenon. A supplementary investigation brought up valuable information through an in silico study of QSI-based therapies, focused on EPS, showing that bacterial spread decreases and demonstrating that it is a good option to continue within new research phases [97].

## 9. Conclusions and Perspectives

Even though information on the impact of QSIs has been obtained through the use of synthetic and natural inhibitors, we are assured this is an adequate anti-infective strategy against bacterial pathogens such as *E. coli* and *Salmonella* spp. In addition, the information in this review is novel due to the representations of crystallization studies in a graphic form designed in PyMOL, using molecular docking models of QSIs in *E. coli* (transcriptional repressor LsrR and A1-2 interactions) and *Salmonella* (LsrR and SdiA interactions), according to the information reported in the Protein Data Bank (PDB). The models are very useful for future studies in which QSI and microorganisms could be proposed and selected. Consequently, it can be considered a promising alternative to the use of traditional antibiotics for the attenuation of bacterial virulence, thus allowing the creation of new anti-QS strategies. Knowledge of the different alternatives of action of QSIs has been used by different studies and research projects carried out by biotechnology companies, confirming how to decrease or attenuate the virulence of bacterial pathogens. These will allow scientists in the future to create more complete and effective anti-QS strategies through molecular tools and, in turn, to propose inhibitors to be used for virulence phenotypes in *E. coli* and *Salmonella* spp. Finally, the mechanisms and genes involved in QSI resistance are not yet known, so it will be interesting to investigate them in the future. Today, the clinical application of QSIs is a novel proposal, and the recommended anti-QS therapies hold challenges and limitations. 

## Figures and Tables

**Table 1:** Natural QS inhibitors in *E. coli* and *Salmonella*.

Natural QSI	Microorganism	Effect on QS-Regulated Process	In Vitro/In Vivo Experiments	Reference
Grape seed extract Reduction in AI activity and synthesis	*E. coli* (STEC), *E. coli* (VTEC), and *E. coli* (EAEC)	Reduces the production of the flagellum and inhibits the production of the Shiga toxin	In vitro	[8,41,48]
Extracts of *Melia dubia* bark	*E. coli* (EHEC)	Hemolysin suppression, effect on mobility-type swarming, and prevents the formation of biofilm	In vitro	[37]
Thymol-carvacrol-chemotype (I and II) oils from *Lippia origanoides* and *Thymus vulgaris* oil	*E. coli*	Prevents the formation of biofilm	In vivo: VERO cell line	[49]
Broccoli extracts, basil, oregano, thyme, rosemary, ginger, and turmeric	*E. coli* (EHEC)	Reduces AI-2 synthesis, with effects on mobility-type swarming and virulence	In vitro	[47,50,51]
Punicalagin from a component of pomegranate rind	*S. enteritidis*	Effect on mobility-type swimming and swarming	In vivo: human colonic HT-29 cell line	[52]
Star anise	*S. typhimurium*	Prevents mobility and biofilm formation	In vitro	[53]
Organic acids: acetic acid, citric acid, and lactic acid	*S. typhimurium* and *E. coli*	Decreases the production of AI-2 and biofilm formation	In vitro	[18,19]
Grapefruit juice/furocoumarin	*S. typhimurium*	Inhibition of AI-2 activity	In vitro	[54]

**Table 2:** Synthetic QS inhibitors in *E. coli* pathogroups.

Synthetic QSI	Microorganism	Effect on QS Regulated Process	In Vitro/In Vivo Experiments	Reference
Thiophene inhibitor (TF101)	*E. coli*	Reduces virulence and prevents the formation of biofilm, cytotoxicity, and the expression of *fimH* and *lsrB*	In vitro and in vivo: Caco-2 cell line	[10]
Furanone	*E. coli*	Prevents AI-2 synthesis	In vivo: mice tissues of lung, liver, spleen, and kidney C57BL/6 cell line	[57]
*Cinnamomum verum* bark essential oil or combination with piperacillin	*E. coli* (multidrug-resistant)	Prevents the formation of biofilm	In vitro	[56,58]
Chitosan	*E. coli* (UPEC)	Reduces virulence, prevents the formation of biofilm, and reduces mobility	In vitro	[59]
Fructose-furoic acid ester	*E. coli* (UPEC)	Decreases toxicity and biofilm production	In vivo: kidney carcinoma A498 cell line	[40]
Limonene nanoemulsion	*E. coli* (EHEC)	Reduces AI-2 synthesis, effect on mobility-type swimming and swarming, and suppression of curli and the extracellular polymeric substance (EPS)	In vivo	[56]
N-phenyl-4-phenylamino-thioxomenthyl amino-benzenesulfonamide	*E. coli* (EHEC) and *S. typhimurium*	Inhibition of QseC-mediated activation of virulence gene expression	In vivo: mice strain 129 × 1/SvJ	[21]
